# Back pain in pregnant women attending an antenatal clinic in KwaZulu-Natal, South Africa

**DOI:** 10.4102/hsag.v26i0.1507

**Published:** 2021-07-30

**Authors:** Carmen Hawker, Laura O’Connor, Poovendhree Reddy, Firoza Haffejee, Maureen N. Sibiya, Dorinda Borg, Shanaz Ghuman, Thembilihle S.P. Ngxongo, Nalini Govender

**Affiliations:** 1 Department of Chiropractic, Faculty of Health Sciences, Durban University of Technology, Durban, South Africa; 2 Department of Community Health Studies, Faculty of Health Sciences, Durban University of Technology, Durban, South Africa; 3 Department of Basic Medical Sciences, Faculty of Health Sciences, Durban University of Technology, Durban, South Africa; 4 DVC Teaching and Learning, Durban University of Technology, Durban, South Africa; 5 Department of Somatology, Faculty of Health Sciences, Durban University of Technology, Durban, South Africa; 6 Department of Nursing, Faculty of Health Sciences, Durban University of Technology, Durban, South Africa

**Keywords:** pregnancy, back pain, prevalence, South Africa, risk factors

## Abstract

**Background:**

Back pain is not uncommon in pregnant women, but it is often under-reported and can be disabling. International studies report a high prevalence of back pain, especially in the last trimester. Little is known about the prevalence of back pain in South African pregnant women.

**Aim:**

To determine the prevalence and risk factors of back pain in a cohort of pregnant women

**Setting:**

Public primary healthcare clinics and the eThekwini municipality of KwaZulu-Natal (KZN), South Africa

**Methods:**

A descriptive cohort design was used to survey pregnant women (*n* = 303) over the course of their pregnancy. Data were collected at the first antenatal visit and again in the third trimester. Participants gave consent and ethical clearance was obtained from an institutional research ethics committee, from the eThekwini Health District and KZN Provincial Department of Health.

**Results:**

The respondents were young Black African women (mean age of 25.8 (± 6.0), who were mostly unemployed (70.7%), and resided in a resource poor setting. Back pain prevalence at the first antenatal visit and the third trimester was 12.4% (*n* = 35) and 10.9% (*n* = 5), respectively. This condition was associated with carrying water and residing in a hostel or an employee’s property. Being single was associated with less risk for developing back pain.

**Conclusion:**

The prevalence of back pain was low in this cohort of women, yet it resulted in a negative impact on the women’s ability to cope with daily life.

**Contribution:**

This is one of the first studies to describe back pain in a South African pregnant population.

## Introduction

Back pain is a significant health and economic problem affecting a large proportion of the population. It has a high disability rate with a severe impact on both the society and the individual (Docking et al. 2011). It is more common in women than men, attributable to hormones, pain sensitivity, as well as to social and psychological factors (Sencan et al. 2017). Back pain is one of the most common problems affecting women of childbearing age, and will affect half of all women at some stage during their pregnancy (Abebe et al. 2014; Usman et al. 2017). Many women will experience their first episode of back pain during pregnancy (Sencan et al. 2017) whilst some will experience persisting back pain post-partum (Abebe et al. 2014; Ayanniyi et al. 2006; Bergström, Persson & Mogren 2016). For many women, there is an expectation that whilst being pregnant, life continues normally (Mota et al. 2015). Back pain in pregnancy has attracted the attention of researchers globally (Jimoh et al. 2013; Ramachandra et al. 2015) and according to Ayanniyi et al. (2006), there is no doubt that back pain is one of the most common complications associated with pregnancy and is often accepted as inevitable (Stuber & Smith 2008).

The prevalence of pregnancy-related back pain is said to vary from 20% to 90% (Bergström et al. 2016; Usman et al. 2017). Factors such as previous back pain, strenuous work, back pain in previous pregnancies, increasing gravidity, as well as demographic and psycho-social factors (Charpentier et al. 2012) have been associated with increased risk. Most women fail to seek help until pain interferes with their daily lives (Sencan et al. 2017). Women are often encouraged to believe that their symptoms are temporary and self-limiting (Abebe et al. 2014; Ayanniyi et al. 2006), with their complaints often being dismissed as ‘normal aches and pains of pregnancy’ (Usman et al. 2017). Despite the high occurrence of pregnancy-related back pain, it remains a trivial aspect of pregnancy healthcare (Quaresma et al. 2010). The condition negatively impacts a woman’s functioning and well-being, often resulting in sick leave, altered activities of daily living, deteriorating quality of life and ability to work (Mota et al. 2015). The characteristics of daily activities and their contribution to musculoskeletal (MSK) disorders, especially in pregnancy are not well-documented (Beaucage-Gauvreau, Dumas & Lawani 2012).

Women in developing countries are subject to intensive activities of daily living. West African women participate in laborious daily activities, ranging from farm work, drawing water from wells and carrying water for long distances, to commercial activities that require carrying heavy loads on their heads (Beaucage-Gauvreau et al. 2012). Intensive farm work and heavy weight-lifting were associated with the increased severity of back pain in Lesotho women (Worku 2000). South Africa has a diverse society, with various social, economic and environmental challenges. There is a high unemployment rate (29.8%) (Statistics South Africa 2011) with a large percentage of female-headed households (Dungumaro 2008). Approximately, 84% of the population depends on the public healthcare system, which is afflicted with human resource shortages and limited resources. This burden negatively impacts quality healthcare for the majority of the population (Benatar 2013). The current management of back pain at primary healthcare (PHC) level has been reported to be ineffective with little conformity to guidelines (Major-Helsloot et al. 2014). The 2000 Millennium Development Goals focus on maternal health, and integrated antenatal care has been posited as a mechanism to reach this target (Fowkes et al. 2016). In a country where the burden of infectious disease outweighs that of non-communicable disorder, there is often little focus on the latter. International literature on the effects of back pain in pregnancy is well established, and has attracted much attention. Moreover, limited data exist on the impact of back pain in pregnant women in developing countries (Charpentier et al. 2012), including South Africa. In light of this and the unique context of South Africa, the current study aimed to provide a description of back pain experienced by a cohort of pregnant women residing in a resource poor setting.

## Research methods and design

### Study design

A descriptive cohort design was used to survey pregnant women (*n* = 303), with the aim to determine back pain characteristics, over the course of their pregnancy. Data were collected at the first antenatal visit and again in the third trimester. The study was conducted between October 2015 and October 2016.

### Study location

This study was conducted at the antenatal clinic (ANC) in a PHC, in Umkhumbane, eThekwini District Municipality, KZN. There are 16 beds in a Medical Outpatient Unit run by the Provincial and eThekwini Municipality.

### Study population

Women presenting at the clinic for their first ANC visit were invited by a trained research nurse, who was fluent in isi-Zulu and English, to participate in the survey. Informed consent was obtained prior to enrolment. All willing participants (*n* = 303) completed the surveys, either in isi-Zulu or English, whilst they waited for their appointment at the clinic.

### Sources of data

At the first antenatal visit, data were collected using the patients’ clinical records, a socio-demographic questionnaire (developed by Napier et al. [2009]) and an epidemiological questionnaire. Information obtained from the patients’ clinical records included height, weight and HIV status. The socio-demographic questionnaire provided data such as demographic characteristics, place of residence, accommodation, employment type and status and education level. The epidemiological questionnaire collected MSK data related to back pain prior to and during pregnancy. The questionnaires were designed by similar studies (Kristiansson, Svärdsudd & Von Schoultz 1996; Skaggs et al. 2007); the modified Nordic Pain Questionnaire (Crawford 2007) and the Bournemouth pain questionnaire (BQ)(Bolton & Breen 1999). Additional items added to the modified Nordic Pain Questionnaire included ‘when did the pain start?’, ‘is your pain: mild, moderate or severe’, ‘has your pain interfered with your ability to perform your daily activities, such as gardening, house work, etc.’ and ‘has the pain affected your ability to work?’. The questionnaire was assessed for content and face validity through an expert group discussion and pilot tested for suitability for the target population. It was re-administered at the third trimester, but excluded questions relating to pre-pregnancy back pain.

### Statistical analysis

The IBM statistical package, SPSS version 24 (IMB Analytics) was used to analyse the data. A *p*-value of less than 0.05 indicated statistical significance. Descriptive statistics were used to describe the data. All relationships investigated were categorical in nature, thus Chi-square test and or Fischer’s exact tests were utilised.

### Ethical considerations

Ethical clearance was given by the Durban University of Technology’s Institutional Research Ethics Committee (reference number: REC106/17), eThekwini Health District and KZN Provincial Department of Health prior to commencement of data collection.

## Results

The mean participant age was 25.8 (± 6.0) with the majority being between 18 and 24 years (44.6%, *n* = 135). All participants were black Africans (*n* = 303), with 82% having a marital status of single (*n* = 246). Unemployment was 70.7% (*n* = 212), with 77% of participants having obtained a secondary level of education (*n* = 228). More than half of the participants resided in a town, city or township (54.1%, *n* = 160), either rented or owned their house and flat (36.6%, *n* = 107) or lived with a friend or relative (31.2%, *n* = 91). The body mass index (BMI) of the women showed that 32.4% of them (*n* = 82) were overweight and 26.9% of them (*n* = 68) were obese. Furthermore, 38.7% were nulliparous (*n* = 98) and 51.2% were multigravida (*n* = 155). Of the total 303 recruited, only 47 women returned to the clinic at trimester three for continued antenatal care.

Back pain prevalence was reported pre-pregnancy as 5.6% (*n* = 17), this increased to 12.4% (*n* = 35) at the first antenatal visit (*n* = 283), with 10.9% (*n* = 5) reporting suffering back pain at the third trimester visit (*n* = 47). At the first antenatal visit, the most common area affected was the low back pain (LBP), with the majority reporting that the pain started within 2 weeks of the first antenatal visit (41.2%, *n* = 7). The pain was described as mild in nature (47.6%, *n* = 10) and that it had little impact on their activities of daily living (54.6%, *n* = 6). At the third trimester, the participants reported experiencing LBP only, of these participants, 66.7% (*n* = 2) reported that the pain started by the first antenatal visit and they described the pain as mild to moderate. In addition, 40% (*n* = 2) of those who suffered LBP in the third trimester indicated that it affected their activities of daily living (Table 1).

**TABLE 1:** Location, duration, severity and impact of back pain in the first and third trimester.

Variables	First antenatal visit						Third trimester visit	
	NP (*n* = 8)		M/UBP (*n* = 14)		LBP (*n* = 24)		LBP (*n* = 5)	
	*n*	%	*n*	%	*n*	%	*n*	%
**Duration (*n* = 7)**								
< 2 weeks	1	14.3	6	66.7	7	41.2	-	-
3–8 weeks	3	42.9	1	11.1	3	17.6	-	-
3–6 months	-	-	-	-	2	11.8	2	66.7
> 6 months	3	42.9	2	22.2	5	29.4	1	33.3
**Severity (*n* = 7)**								
Mild	3	42.9	3	25.0	10	47.6	2	40.0
Moderate	4	57.1	7	58.3	9	42.9	2	40.0
Severe	-	-	2	16.7	2	9.5	1	20.0
**Impact on activities of daily living (*n* = 7)**								
Yes	3	42.9	5	45.5	6	31.6	2	40.0
No	4	57.1	6	54.5	13	68.4	3	60.0

NP, neck pain; M/UBP, mid/upper back pain; LBP, low back pain.

Many of the respondents partook in daily activities such as carrying water (30%, *n* = 88) and house work (73%; *n* = 214) with very few doing manual labour (7.2%, *n* = 21) or gardening (4.4%, *n* = 13). The only activity of daily living that was associated with back pain pre-pregnancy (*p* < 0.001) and at the first antenatal visit (*p* = 0.009) was carrying water (Table 2).

**TABLE 2:** Activities of daily living and back pain prevalence.

Variables	Back pain prevalence																				
	Pre-pregnancy							First antenatal visit							Third trimester visit						
	Total	%	Yes	%	No	%	*p*	Total	%	Yes	%	No	%	*p*	Total	%	Yes	%	No	%	*p*
**Carry water**																					
Yes	88	30.0	13	30.0	75	25.6	0.001*	83	29.7	17	6.1	66	23.7	0.009	14	32.6	1	2.3	13	30.2	1.000
No	205	70.0	3	1.0	202	68.9		196	70.3	17	6.1	179	64.2		29	67.4	4	9.3	25	58.1	
Total	293	100.0	16	5.5	277	94.5		279	100.0	34	12.2	245	87.8		43	100.0	5	11.6	38	88.4	
**Gardening**																					
Yes	13	4.4	0	0.0	13	4.4	1.000	12	4.3	1	0.4	11	3.9	1.000	2	4.7	0	0.0	2	4.7	1.000
No	280	95.6	16	5.5	264	90.1		267	95.7	33	11.8	234	83.9		41	95.3	5	11.6	36	83.7	
Total	293	100.0	16	5.5	277	94.5		279	100.0	34	12.2	245	87.8		43	100.0	5	11.6	38	88.4	
**House work**																					
Yes	214	73.0	9	3.1	205	70.0	0.147	200	71.7	22	7.9	178	63.8	0.416	33	76.7	4	9.3	29	67.4	1.000
No	79	27.0	7	2.4	72	24.6		79	28.3	12	4.3	67	24.0		10	23.3	1	2.3	9	20.9	
Total	293	100.0	16	5.5	277	94.5		279	100.0	34	12.2	245	87.8		43	100.0	5	11.6	38	88.4	
**Manual labour**																					
Yes	21	7.2	2	0.7	19	6.5	0.321	19	6.8	2	0.7	17	6.1	1.000	3	7.0	0	0.0	3	7.0	1.000
No	272	92.8	14	4.8	258	88.1		260	93.2	32	11.5	228	81.7		40	93.0	5	11.6	35	81.4	
Total	293	100.0	16	5.5	277	94.5		279	100.0	34	12.2	245	87.8		43	100.0	5	11.6	38	88.4	

* , *p* ≤ 0.05

Participants were asked, at the first antenatal and third trimester visit, about the impact of their back pain, on their ability to socialise, their frame of mind, the ability to cope with the pain and if the pain raised enough concern for them to seek treatment. The majority of the participants, at the first antenatal visit reported that the back pain had little impact on their lives (Table 3), whereas those returning for the third trimester visit showed a decreased ability to cope with the pain (57.9%, *n* = 11). However, only one person sought treatment (7.1%).

**TABLE 3:** Impact of back pain on the participants as reported at the first and third trimester visits.

Question	First trimester		Third trimester	
	*n*	%	*n*	%
**Pain prevented spending time with family and friends**				
Yes	13	19.4	3	14.3
No	54	80.6	18	85.7
**Pain made me concerned about health**				
Yes	27	40.3	7	35
No	40	59.7	13	65
**Pain made me feel sad or depressed**				
Yes	20	29.9	4	21.1
No	47	70.1	15	78.9
**I am able to cope with the pain**				
Yes	53	80.3	8	42.1
No	13	19.7	11	57.9
**I sought treatment for the pain**				
Yes	21	31.3	1	7.1
No	46	68.7	13	92.9

No significant relationships were found between back pain prevalence and socio-demographic characteristics of the participants except for their type of accommodation. Staying in a hostel or at an employee’s property was associated with pre-pregnancy back pain (*p* = 0.030), whilst being single was associated with less risk for developing back pain (*p* = 0.018; odds ratio [OR] = 0.437; CI: 0.233–0.820) (Table 4).

**TABLE 4:** Socio-demographic factors and back pain prevalence.

Socio-demographic characteristics	Back pain prevalence																				
	Pre-pregnancy							First trimester visit							Third trimester visit						
	Total		Yes		No		*p*	Total		Yes		No		*p*	Total		Yes		No		*p*
	*n*	%	*n*	%	*n*	%		*n*	%	*n*	%	*n*	%		*n*	%	*n*	%	*n*	%	
**Age**																					
< 18	17	5.6	0	0.0	17	5.6	0.457	17	6.0	2	0.7	15	5.3	0.762	2	4.3	1	50.0	1	50.0	0.150
18–24	135	44.6	11	3.6	124	40.9		127	44.9	16	5.7	111	39.2		20	43.5	1	5.0	19	95.0	
25–35	126	41.6	5	1.7	121	39.9		117	41.3	16	5.7	101	35.7		21	45.7	2	9.5	19	90.5	
> 36	25	8.3	1	0.3	24	7.9		22	7.8	1	0.4	21	7.4		3	6.5	1	33.3	2	66.7	
Total	303	100.0	17	5.6	286	94.4		283	100.0	35	12.4	248	87.6		46	100.0	5	10.9	41	89.1	
**Marital status**																					
Married‎ or cohabiting	49	16.2	3	1.0	46	15.2	1.000	46	16.3	9	3.2	37	13.1	0.033*	6	13.0	1	2.2	5	10.9	0.634
Single	246	81.5	14	4.6	232	76.8		229	81.2	24	8.5	205	72.7		38	82.6	4	8.7	34	73.9	
Divorced‎ or separated	6	2.0	0	0.0	6	2.0		6	2.1	1	0.4	5	1.8		2	4.3	0	0.0	2	4.3	
Widow or other	1	0.3	0	0.0	1	0.3		1	0.4	1	0.4	0	0.0		0	0.0	0	0.0	0	0.0	
Total	302	100.0	17	5.6	285	94.4		282	100.0	35	12.4	247	87.6		46	100.0	5	10.9	41	89.1	
**Accommodation**																					
Rented or /own	107	36.6	7	2.4	100	34.2	0.030*	98	36.0	13	4.8	85	31.3	0.074	18	42.9	2	4.8	16	38.1	0.766
Relative‎ or friend	91	31.2	4	1.4	87	29.8		87	32.0	10	3.7	77	28.3		13	31.0	1	2.4	12	28.6	
Squatter camp	72	24.7	1	0.3	71	24.3		66	24.3	4	1.5	62	22.8		6	14.3	1	2.4	5	11.9	
Hostel or employees property or other	19	6.5	3	1.0	16	5.5		18	6.6	5	1.8	13	4.8		5	11.9	1	2.4	4	9.5	
Homeless	3	1.0	1	0.3	2	0.7		3	1.1	1	0.4	2	0.7		0	0.0	0	0.0	0	0.0	
Total	292	100.0	16	5.5	276	94.5		272	100.0	33	12.1	239	87.9		42	100.0	5	11.9	37	88.1	
**Employment**																					
Yes	88	29.3	7	2.3	81	27.0	0.280	82	29.2	12	4.3	70	24.9	0.424	16	34.8	1	2.2	15	32.6	0.645
No	212	70.7	10	3.3	202	67.3		199	70.8	22	7.8	177	63.0		30	65.2	4	8.7	26	56.5	
Total	300	100.0	17	5.7	283	94.3		281	100.0	34	12.1	247	87.9		46	100.0	5	10.9	41	89.1	
**Highest level of education**																					
None	4	1.4	0	0.0	4	1.4	0.641	4	1.4	0	0.0	4	1.4	0.799	2	4.4	0	0.0	2	4.4	1.000
Basic education	228	77.0	12	4.1	216	73.0		213	77.2	28	10.1	185	67.0		29	64.4	3	6.7	26	57.8	
Post school education	64	21.6	5	1.7	59	19.9		59	21.4	6	2.2	53	19.2		14	31.1	1	2.2	13	28.9	
Total	296	100.0	17	5.7	279	94.3		276	100.0	34	12.3	242	87.7		45	100.0	4	8.9	41	91.1	

* , *p* ≤ 0.05.

## Discussion

It is estimated that the majority of women will experience some degree of MSK discomfort during pregnancy, of which back pain is most prevalent (Jimoh et al. 2013). Pregnancy-related back pain ranges from 25% to 90%, with more than 50% of pregnant women suffering with LBP (Wang et al. 2004). Studies in Sub-Saharan Africa are lacking in terms of describing back pain in pregnant women, especially in SA. Our study revealed a low prevalence of back pain in the first (12.4%) and third (11.1%) trimesters of pregnancy. This is in contrast to studies conducted in other developing countries such as India (Usman et al. 2017) and Ethiopia (Abebe et al. 2014), where back pain prevalence was 34.3% and 33.2%, respectively. Many factors can influence the prevalence of pregnancy-related back pain. The participants in this study were mostly young Black females experiencing their first pregnancy.

Half of the participants (50.2%) in this study were below the age of 24. Early childbearing is common in SA with a high prevalence of teenage pregnancies (Panday et al. 2009). In non-pregnant populations, back pain prevalence increases from the third decade (Hoy et al. 2012), yet in pregnancy, the relationship between age and back pain is inconclusive. Our study similar to the study by Abebe et al. (2014) found no relationship between pregnancy-related back pain and age. The marital status of the respondents indicated that most females were single (81.5%). Being separated, divorced or widowed has been associated with a higher incidence of back pain possibly because of a lack of support. Being married increases social support, has health benefits and decreases stress with regard to social, financial and living situations (Reblin & Uchino 2008). Although the respondents in this study were mostly single, many (31.2%) resided with a friend or relative who could offer social and financial support because of shared living expenses.

The participants in this study consulted a PHC clinic in a resource poor setting. A lack of education, low income, unemployment and disadvantaged living areas have been reported to predispose females to back pain (Silva et al. 2008). In this study, however, no associations between these variables and back pain prevalence were found, irrespective of the trimester. Low socio-economic status and education are often associated with strenuous work activities (Charpentier et al. 2012). Many of the participants in this study engaged in carrying water (30%) and doing housework (73%). Tasks like carrying water can lead to strain on the body resulting in pain (Geere et al. 2018). Results of this study support this finding, as carrying water was the only common activity of daily living that was associated with back pain. In 80% of households where there is no access to water on the property, women and girls are responsible for its collection (United Nations 2019). Crucial household tasks need to be met daily, irrespective of whether or not a woman is pregnant. The characteristics of daily activities and their contribution to MSK disorders, especially back pain are not well-documented (Beaucage-Gauvreau et al. 2012) and in developing countries where these activities are carried out in combination, a cumulative burden may exist.

Some studies suggest that there is an association amongst back pain in pregnancy, parity and gravida (Mogren & Pohjanen 2005; Mohseni-Bandpei et al. 2009; Mota et al. 2015), our data report no associations as most participants reported this pregnancy as their first pregnancy (38.7%). Obesity has been associated as a predictor of chronic widespread MSK pain (Heuch et al. 2013), and there is a global increase in obesity prevalence, especially in SA. Notably, 59.3% of our participants were identified as being overweight. An earlier study suggest that pregnant women with back pain have a higher BMI compared to those without back pain (Mogren & Pohjanen 2005), however, our data demonstrate no association between BMI and back pain prevalence.

The most common spinal pain experienced by the participants was LBP, similar to other studies (Gorginzadeh, Imani & Safari 2016; Mota et al. 2015; Ramachandra et al. 2015; Sencan et al. 2017). At the first antenatal visit, those respondents experiencing neck and upper or mid back pain reported it to be acute and sub-acute, whilst in contrast to those with LBP reported a more chronic nature to their pain. On an average, the pain was rated as mild to moderate, with less than half reporting that it impacted their activities of daily living. The majority of pregnant women do not seek medical advice for back pain until it interferes with their daily activities (Ramachandra et al. 2015). It is often seen as an inevitable part of pregnancy (Stuber & Smith 2008). This is supported by Usman et al. (2017) who suggested that only a small percentage of pregnant women experienced severe back pain. Back pain is often more common in the latter part of pregnancy as changes from pregnancy become more pronounced. In this study, 57.9% of women in the third trimester reported that they were unable to cope with the pain compared to only 19.7% at the first antenatal visit. Care should be taken in this interpretation because of the low number of women who partook in the third trimester, it does, however, highlight the negative impact back pain can have in pregnancy.

The low return rate (15%) of participants to the clinic for their third trimester visit shows the difficulty in performing pregnancy cohorts in developing countries. Women come to the city to work, fall pregnant and then return to the rural farm areas to deliver their babies. This impacts the ability to track the cohort through the gestational period. Access to adequate ANC care has been a strategy to address the fourth Millennium Development Goal to reduce child mortality. ANC care in developing countries has a major focus on reducing pregnancy-related mortality and morbidity, taking measures to make the gestational period as tolerable as possible for mothers and their families (Gorginzadeh et al. 2016). South Africa has made significant progress with regard to the improvement of maternal health and the reduction of maternal mortality in the last two decades (National Department of Health, Statistics SA, South African Medical Research Council & ICF 2017), yet more work is required.

Current management at PHC levels in SA has been ineffective with no conformity to guidelines (Major-Helsloot et al. 2014). This is one of the first studies, to our knowledge, that has investigated back pain in a pregnant cohort in SA. Despite the low prevalence of back pain in these pregnant women from a resource poor setting, further studies are necessary in similar and different contexts within SA to determine the impact of back pain in pregnancy and to assess other factors such as exercise.

## Conclusion

This study showed a low prevalence of back pain in pregnancy in a South African cohort of women attending a public ANC in a resource poor setting. As pregnancy duration increased, the ability to cope with the back pain decreased, highlighting that even if the prevalence is low, the disability for those who suffer from back pain in pregnancy is great.
